# Transcriptomic profile adaptations following exposure of equine satellite cells to nutriactive phytochemical gamma-oryzanol

**DOI:** 10.1186/s12263-016-0523-5

**Published:** 2016-03-17

**Authors:** K. A. Szcześniak, A. Ciecierska, P. Ostaszewski, T. Sadkowski

**Affiliations:** Department of Physiological Sciences, Faculty of Veterinary Medicine, Warsaw University of Life Sciences - SGGW, Nowoursynowska 159, 02-776 Warsaw, Poland

**Keywords:** Gamma-oryzanol, Equine satellite cells, Differentiation, Muscle, Gene expression

## Abstract

**Background:**

Adult skeletal muscle myogenesis depends on the activation of satellite cells that have the potential to differentiate into new fibers. Gamma-oryzanol (GO), a commercially available nutriactive phytochemical, has gained global interest on account of its muscle-building and regenerating effects. Here, we investigated GO for its potential influence on myogenesis, using equine satellite cell culture model, since the horse is a unique animal, bred and exercised for competitive sport. To our knowledge, this is the first report where the global gene expression in cultured equine satellite cells has been described.

**Methods:**

Equine satellite cells were isolated from *semitendinosus* muscle and cultured until the second day of differentiation. Differentiating cells were incubated with GO for the next 24 h. Subsequently, total RNA from GO-treated and control cells was isolated, amplified, labeled, and hybridized to two-color *Horse Gene Expression Microarray* slides. Quantitative PCR was used for the validation of microarray data.

**Results:**

Our results revealed 58 genes with changed expression in GO-treated vs. control cells. Analysis of expression changes suggests that various processes are reinforced by GO in differentiating equine satellite cells, including inhibition of myoblast differentiation, increased proliferation and differentiation, stress response, and increased myogenic lineage commitment.

**Conclusions:**

The present study may confirm putative muscle-enhancing abilities of GO; however, the collective role of GO in skeletal myogenesis remains equivocal. The diversity of these changes is likely due to heterogenous growth rate of cells in primary culture. Genes identified in our study, modulated by the presence of GO, may become potential targets of future research investigating impact of this supplement in skeletal muscle on proteomic and biochemical level.

**Electronic supplementary material:**

The online version of this article (doi:10.1186/s12263-016-0523-5) contains supplementary material, which is available to authorized users.

## Background

Since horses were domesticated on the Eurasian Steppe over 6000 years ago, they had been selected for strength, speed, and endurance exercise [23]. Nowadays in Europe, working horses are very seldom used for farming purposes; they became exclusively domestic animals exercised for competitive sport. In both equine and human athletes, nutritional ergogenic aids have been used to improve physical ability with an appropriate training regimen. Although training increases physical condition, the ease of taking a nutritional additive to improve training results explains the demand for supplementation, which may increase mechanical energy of work, delay onset of fatigue, and improve neuromuscular coordination [26].

Gamma-oryzanol (GO) is a unique mixture of triterpene alcohol and sterol ferulates present in rice bran oil, a by-product of rice processing. GO has a wide range of potential therapeutically useful activities including prevention of coronary atherosclerosis and anticancerogenic and antidiabetic action [28]; however, in our study, we focused on the anabolic properties of GO.

GO has been shown to be very safe with no major side effects being reported in either animal or human studies and is a popular ergogenic aid approved for commercial use in the USA [17]. Moreover, GO is often used by veterinary practitioners in order to improve muscle mass gain and recovery in horses. It was temporarily accounted as a prohibited substance by the Federation Equestre Internationale (FEI) unit governing rules of fair play in equestrian competition (FEI Veterinary Regulations 11th edition, 1st January 2009) [44].

Despite this, only a few studies of ergogenic use of GO were described in the peer-reviewed literature. In one study, authors looked on the resistance-weight-trained male athletes supplemented with 500 mg of GO daily [19]. However, this study failed to demonstrate any effect of GO on training performance. In a recent study, Eslami et al. [17] revealed that 600 mg/day of GO supplementation changed muscular strength in young healthy males in the 9-week resistance training without any significant alteration in anthropometric measurements. Ostaszewski et al. [49] showed that GO supplementation prevented exercise-induced muscle damage in thoroughbred race horses during 16 weeks of training. Animals received 3 g of GO a day. However, no paper describing GO’s influence on equine satellite cells was published.

Skeletal muscle satellite cells (SC) are mononucleated cells located under the basal lamina of myofiber. They play an indispensable role during adult skeletal muscle regeneration and hypertrophy. SC are quiescent during most of their normal adult life. Upon activation, they proliferate, differentiate, and finally fuse with adjacent muscle fiber or with other satellite cells to form new multinucleated fibers [40]. The procedure of harvesting satellite cells from equine muscles was first described in 1992 [22], but until now, except for that single report, studies concerning equine satellite cells in vitro are scarce. Previous transcriptomic analyses in horses were conducted on peripheral blood cells and muscle samples obtained by muscular biopsy; thus, our report is the first trial where the global gene expression in cultured equine satellite cells has been described [51]. Satellite cells require an activation signal to proliferate and differentiate and can be controlled by a variety of hormones, growth factors, and metabolites. In vitro reports suggest that satellite cells may also be regulated directly by specific dietary components including ergogenic aids [18, 50, 62]. Determining the factors that independently regulate satellite cells is important to understand overall mechanisms involved in muscle growth and regeneration.

This study focused on revealing possible molecular mechanism underlying GO’s influence on skeletal myoblasts. For this purpose, transcriptomic profile analysis of primary culture of equine satellite cells incubated with GO was performed. This in vitro model can help to identify and better understand potential therapeutic to promote muscle regeneration in mammals, among them also in sport horses.

## Methods

### Cell culture media and reagents

The following reagents/materials were used during cell culture: GO powder was purchased from TCI Chemicals, USA; *Penicillinum crystalicum* was purchased from Polfa Tarchomin, Poland; and Dulbecco’s Modified Eagle’s medium (DMEM) (1×) with Glutamax, fetal bovine serum (FBS), horse serum (HS), and antibiotics (AB), penicillin-streptomycin and amphotericin B, were purchased from Gibco, Life Technologies, USA. Phosphate-buffered saline (PBS), protease from *Streptomyces griseus*, and DMSO were purchased from Sigma-Aldrich, USA. Primaria tissue culture flasks (25 and 75 cm^2^) and Collagen I Cellware six-well plates were purchased from Becton Dickinson, USA.

### Animals and muscle samples

Samples of *semitendinosus* muscle were collected from six horses (6-month-old, healthy colts), aseptically in a commercial abattoir during routine slaughter. Next, they were dissected free of connective and adipose tissues to minimize contamination with adipocytes and fibroblasts. The tissue was sliced to pieces, washed four times in PBS with gradually decreasing antibiotic concentration (40,000 units (U) *Penicillinum crystalicum*/100 ml PBS, 20,000 U *Penicillinum crystalicum*/100 ml PBS), suspended in sterile FBS with addition of 10 % DMSO, gradually cooled down to −80 °C, and then stored in liquid nitrogen until isolation.

### Satellite cell isolation, cell culture, and experimental design

Since the purpose of the study was to evaluate the impact of GO on in vitro differentiation, primary satellite cell cultures from *semitendinosus* muscle of all horses were isolated and assessed for cell viability (MTT assay) and fusion index (data not shown). For further analysis, the cell line with the best scores was chosen.

**Fig. 1:**
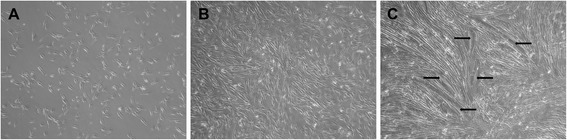
Cultured equine satellite cells: sixth day of proliferation, 30 % confluence (**a**); 10th day of proliferation, 80 % confluence (**b**); and 48 h of differentiation (**c**). Myotubes are marked with *arrows*

**Fig. 2:**
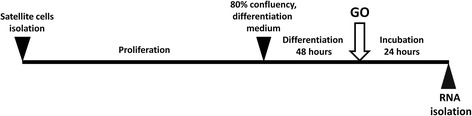
Experiment design. Equine SC were cultured until reaching 80 % of confluence, and then proliferation medium was replaced by differentiation medium. After the second day of differentiation, cells were incubated for 24 h with GO. Following the GO treatment, differentiating cells were scraped and stored at −80 °C until further analysis

To isolate equine satellite cells, six samples of *semitendinosus* muscle were thawed in water bath in 37 °C, centrifuged, and washed three times with PBS with *Penicillinum crystalicum*. Then, each sample was incubated (1.5 h) with DMEM/AB/protease from *S. griseus* (pH 7.3) and sieved in order to separate tissue debris. The filtrates were centrifuged three times, resuspended in proliferation medium (10 % FBS/10 % HS/DMEM/AB), and transferred to polypropylene Petri culture disks. To minimize possible fibroblast contamination, 1.5-h pre-plating was used. Subsequently, supernatant containing myoblasts was transferred to Primaria culture flasks. The growth medium was changed every 2 days. On the 10th day of proliferation, cells were trypsinized and counted by Scepter Cell Counter (Merck Millipore, Germany). Equal number of cells (30,000) from each isolation was transferred to separate the well of Collagen I Cellware six-well plates. After obtaining 80 % of confluence, proliferation medium was replaced by differentiation medium (2 % HS/DMEM/AB). Figure 1 presents equine SC at various stages of growth. At the 48th hour of differentiation, 0.125 μM GO was added and cells were incubated for 24 h. Because GO is insoluble in water, DMSO (0.04 μl/ml) was used as a vehicle. The concentration of GO has been chosen on the basis of assessing cell viability with 3-(4,5-dimethylthiazol-yl)-2-5-diphenyltetrazolium bromide (MTT) colorimetric assay (data not shown). Control medium contained DMSO in the same dose as in GO-treated cells. Following the GO treatment, medium from each plate was discarded and plates were stored at −80 °C until further analysis. Cell culture’s schema is illustrated in Fig. 2.

### RNA isolation, validation, labeling, and hybridization

Total RNA (from GO-treated and control groups, *n* = 6) was isolated according to the protocol supplied with the miRNeasy Mini Kit (Qiagen, USA). RNA quantity was measured spectrophotometrically using NanoDrop (NanoDrop Technologies, USA). The analysis of the final RNA quality and integrity was performed with BioAnalyzer 2100 (Agilent Technologies, USA). To ensure optimal microarray data quality, four RNA samples with the highest RIN ≥ 9.2 were included into the analysis.

Analysis of gene expression profile was performed using *Horse Gene Expression Microarray*, 4 × 44 K (*n* = 4) (Agilent Technologies, USA). Low Input Quick Amp Labeling Kit (Agilent Technologies, USA) was used to amplify and label total RNA (100 ng) to generate complementary RNA (cRNA). On each two-color microarray, 825 ng of cRNA from GO-exposed cells (labeled by Cy5, *n* = 4) and 825 ng of cRNA from control cells (labeled by Cy3, *n* = 4) were hybridized to the arrays (Gene Expression Hybridization Kit, Agilent Technologies, USA) according to the manufacturer’s protocol.

RNA Spike-In Kit (Agilent Technologies, USA) was used as an internal control to efficiently monitor microarray workflow for linearity, sensitivity, and accuracy. Acquisition and analysis of hybridization intensities were performed using the Agilent DNA microarray scanner and Agilent Feature Extraction software 10.7.3.1 according to the standard manufacturer’s procedures and Linear Lowess normalization (Cy5/Cy3 dye bias compensation).

The statistical analysis was performed using Gene Spring 13 software (Agilent Technologies, USA) with default setting for two-color microarrays. The estimated significance level (*p* value) was corrected for multiple hypotheses testing using Benjamini and Hochberg false discovery rate (FDR) adjustment. Messenger RNAs (mRNAs) with FDR ≤ 0.05 and fold change (FC) ≥1.3 were selected as significantly differentially expressed.

The microarray experiment was performed according to MIAME guidelines [6]. The data discussed in this publication have been deposited in NCBI’s Gene Expression Omnibus [16] and are accessible through GEO Series accession number GSE73730 (http://www.ncbi.nlm.nih.gov/geo/query/acc.cgi?acc=GSE73730).

### cDNA synthesis and quantitative real-time PCR

To verify microarray results, the quantitative real-time polymerase chain reaction (qPCR) method was applied. The sequences of verified genes, complementary to those on microarrays, were obtained from Ensembl database. Primers were designed using Primer-Blast software (NCBI database) and then checked for secondary structures using Oligo Calculator (http://www.basic.northwestern.edu/biotools/oligocalc.html). The secondary structures of the amplicon were examined using m-fold Web Server (http://mfold.rna.albany.edu/?q=mfold). The sequences of the primers are listed in Table 1. The primers were purchased from Oligo IBB (Polish Academy of Science, Warsaw, Poland). Each primer pair was quality tested to ensure that a single product was amplified (dissociation curve analysis) and that there was no primer-dimer coupling.

**Table 1:** Primers used for real-time qPCR; full names of genes are available in Table 2

No.	Gene symbol	Forward primer	Reverse primer	Annealing temperature (°C)	Product length (bp)
1.	*bdnf*	CCCCATGAAAGAAGCAAACG	TACAAGTCCGCGTCCTTACT	60	213
2.	*btg1*	GGCTCCATCTGTGTGCTGTA	GCCCACCCAAAGCAAAACTC	60	227
3.	*gja1*	TGCTGCGAACCTACATCATC	CGATGACGTTCAAGGCAAGA	60	255
4.	*igf1*	CAGAAGCAATGGGAAAAATCA	CGTGGGCTTGTTGAAATAAAA	62	242
5.	*itpr2*	CTGTGGGTATTCGGCCATTT	GCCACGATTTCCGACAAAAC	60	143
6.	*mllt3*	GGAACCGAAACCCATGTCAA	GGGCTTTTTGTCAGCAGAAC	60	220
7.	*mstn*	GCCTGGAAACAGCTCCTAAC	GTCGTCGCGTGGTAATCATC	60	151
8.	*myf5*	GGAGACGCCTGAAGAAAGTC	CCGGCAGGCTGTAGTAATTC	60	171
9.	*myog*	CTCGCTCACCTCCATTGTG	CAGTTGGGCATGGTTTCATC	60	78
10.	*tgfb2*	AGTACTACGCCAAGGAGGTT	TAGGCGGGATGGCATTTTCC	60	72
11.	*gapdh*	GTTTGTGATGGGCGTGAACC	GTCTTCTGGGTGGCAGTGAT	60	198

One microgram of total RNA from GO-treated and control samples (*n* = 6) was reverse transcribed using Trancription First-Strand cDNA Synthesis Kit (Roche, USA). All analyses were performed on individual samples of total RNA using a SensiFAST SYBR lo-ROX Kit (Blirt, Bioline, Poland) following the manufacturer’s protocol. Assays for each gene were conducted in duplicate in a Stratagene Mx3005p thermal cycler according to the following protocol: pre-incubation for 2 min at 95 °C and amplification (40 cycles) containing denaturation at 95 °C for 5 s and annealing at a temperature specified in Table 1 for 15 s. Dissociation curve setting was as follows: denaturation at 95 °C for 0 s, annealing at a temperature specified in Table 1, continuous melting up to 95 °C for 0 s (slope = 0.1 °C/s), and cooling at 40 °C for 30 s. *Gapdh* was used as a reference gene. The relative expression of the target gene was calculated according to the following formula:

$$ \Delta \Delta \mathrm{C}\mathrm{T}=\Delta \mathrm{C}\mathrm{T}\left(\mathrm{sample}\right)-\Delta \mathrm{C}\mathrm{T}\left(\mathrm{control}\right) $$

where ΔCT is the difference in CT between the targeted gene and reference control. Results were calculated as 2^−ΔΔCT^ [38] using GenEx 6.0 (MultiD Analyses, Sweden). The amplification efficiency (*E* = 10^(−1/slope)^ − 1) was determined by performing a comparative quantitation standard curve and was >0.9 for each target gene and the reference gene. Standard curves were generated using a four-point 1:10 dilution series starting with cDNA representing 10 ng of input total RNA. qPCR analysis has been conducted according to a standardized approach [10].

### Functional analysis

The list of significantly modulated by GO genes was analyzed by Functional Analysis tool in the Database for Annotation, Visualization and Integrated Discovery (DAVID version 6.7) (da Huang et al. 2009) in order to identify their annotations in gene ontology divided into three functional classes: biological processes, cellular components, and molecular functions. Gene ontology enrichment was calculated by EASE score corrected for multiple hypotheses testing using Benjamini and Hochberg FDR.

Relationships between all differentially expressed genes (microarray and RT-PCR) were visualized with Pathway Studio’s Build Pathway functionality (Elsevier, USA) which is based on the wave-propagation algorithm developed for the navigation through complex networks. In this analysis, Find Direct Links/All Objects Directions Algorithm was used. Protein entity type and direct regulation, regulation, expression, and promoter-binding relationships were selected in the analysis. Such created graph was expanded with five muscle-related biological processes.

## Results

### The number of differentially expressed genes

Comparison of gene expression between GO-treated and control cells revealed statistically significant (FDR ≤ 0.05, FC ≥ 1.3) differences in the expression of 97 transcripts. Sequences of differentially expressed probes not annotated by microarray manufacturer were compared with the NCBI nucleotide databases (using blastn) to assign a gene name. Probes not matching in 100 % with any eukaryotic mRNA/gene were excluded from gene lists used for functional analysis. That resulted in 58 identified, unduplicated, transcript IDs including 17 up- and 41 down-regulated genes in GO vs. control group. The list of identified transcripts can be found in Table 2. All array data are plotted and shown in Additional file 1.

**Table 2:** List of differentially expressed genes in GO-treated vs. CTRL equine satellite cells—microarray analysis (FDR ≤ 0.05, fold change ≥1.3, *n* = 4)

No.	Gene symbol	Regulation	FC	Corr. *p* value	Description	Accession number
1.	*il-1r2*	Up	3.1	0.040	Interleukin-1 receptor type II	[NM_001081816]
2.	*efhc2*	Up	3.0	0.049	EF-hand domain (C-terminal) containing 2	[ENSECAT00000017638]
3.	*tcrd*	Up	2.2	0.050	T cell receptor delta	[L38389]
4.	*tfap2e*	Up	1.8	0.039	Transcription factor AP-2 epsilon (activating enhancer-binding protein 2 epsilon)	[ENSECAT00000003183]
5.	*cdca7*	Up	1.7	0.027	Cell division cycle associated 7	[ENSECAT00000012017]
6.	*f2rl2*	Up	1.6	0.048	Coagulation factor II (thrombin) receptor-like 2	[ENSECAT00000010830]
7.	*rcor2*	Up	1.5	0.026	REST corepressor 2	[ENSECAT00000024786]
8.	*lrrc16a*	Up	1.5	0.049	Leucine-rich repeat containing 16A	[ENSECAT00000026357]
9.	*mapk14*	Up	1.5	0.037	Mitogen-activated protein kinase 14	[XM_005604060]
10.	*fezf2*	Up	1.4	0.029	FEZ family zinc finger 2	[ENSECAT00000022391]
11.	*colec12*	Up	1.4	0.026	Collectin subfamily member 12	[XM_005604060]
12.	*pir*	Up	1.4	0.038	Pirin (iron-binding nuclear protein)	[ENSECAT00000014292]
13.	*gja1*	Up	1.4	0.048	Gap junction protein 1	[ENSECAT00000005498]
14.	*itpr2*	Up	1.4	0.05	Inositol 1,4,5-trisphosphate receptor, type 2	[ENSECAT00000009891]
15.	*tmem107*	Up	1.3	0.045	Transmembrane protein 107	[ENSECAT00000014966]
16.	*steap1*	Up	1.3	0.045	6 transmembrane epithelial antigen of the prostate (metalloreductase)	[ENSECAT00000024982]
17.	*alcam*	Up	1.4	0.045	Activated leukocyte cell adhesion molecule	[XM_005602026]
18.	*musk*	Down	−1.3	0.042	Muscle, skeletal, receptor tyrosine kinase	[ENSECAT00000010687]
19.	*exoc6*	Down	−1.3	0.044	Exocyst complex component 6	[ENSECAT00000025821]
20.	*sept6*	Down	−1.3	0.036	Septin 6	[XM_005614469]
21.	*mdh2*	Down	−1.3	0.039	Malate dehydrogenase 2, NAD (mitochondrial)	[NM_001195526]
22.	*nexn*	Down	−1.3	0.039	Nexilin (F-actin-binding protein)	[ENSECAT00000016039]
23.	*setd7*	Down	−1.3	0.026	SET domain containing lysine methyltransferase 7	[ENSECAT00000020466]
24.	*tmem47*	Down	−1.3	0.045	Transmembrane protein 47	[XM_008544808]
25.	*bdnf*	Down	−1.3	0.027	Brain-derived neurotrophic factor	[NM_001081787]
26.	*snca*	Down	−1.3	0.038	Synuclein, alpha (non A4 component of amyloid precursor)	[ENSECAT00000016509]
27.	*actg2*	Down	−1.4	0.045	Actin, gamma 2, smooth muscle, enteric	[ENSECAT00000020181]
28.	*btg1*	Down	−1.4	0.039	B cell translocation gene 1, antiproliferative	[ENSECAT00000022009]
29.	*znf423*	Down	−1.5	0.045	Zinc finger protein 423	[ENSECAT00000000207]
30.	*aqp1*	Down	−1.5	0.027	Aquaporin 1	[XM_003364834]
31.	*bcl2l11*	Down	−1.5	0.035	BCL2-like 11 (apoptosis facilitator)	[ENSECAT00000023850]
32.	*nr2f2*	Down	−1.5	0.040	Nuclear receptor subfamily 2, group F, member 2	[ENSECAT00000008488]
33.	*pak1*	Down	−1.5	0.050	CDC42 effector protein (Rho GTPase binding) 3	[ENSECAT00000019645]
34.	*cryab*	Down	−1.5	0.035	Crystallin, alpha B	[ENSECAT00000012936]
35.	*abra*	Down	−1.5	0.045	Actin-binding Rho-activating protein	[ENSECAT00000016765]
36.	*capn6*	Down	−1.5	0.038	Calpain 6	[ENSECAT00000011583]
37.	*herpud1*	Down	−1.5	0.045	Homocysteine-inducible, endoplasmic reticulum stress-inducible (ubiquitin-like domain member 1)	[JL623642]
38.	*tmcc3*	Down	−1.6	0.033	Transmembrane and coiled-coil domain family 3	[ENSECAT00000012141]
39.	*tbc1d8*	Down	−1.6	0.027	TBC1 domain family, member 8 (with GRAM domain)	[ENSECAT00000013116]
40.	*kiaa1958*	Down	−1.6	0.045	Protein-coding gene	[ENSECAT00000008002]
41.	*neo1*	Down	−1.6	0.038	Neogenin 1	[ENSECAT00000026822]
42.	*lrrn1*	Down	−1.6	0.045	Leucine-rich repeat neuronal 1	[ENSECAT00000002791]
43.	*ctrc*	Down	−1.6	0.044	Chymotrypsin C (caldecrin)	[XM_008540961]
44.	*itgb1bp2*	Down	−1.6	0.048	Integrin beta 1-binding protein (melusin) 2	[ENSECAT00000016364]
45.	*stk17b*	Down	−1.6	0.039	Serine/threonine kinase 17b (apoptosis-inducing)	[JL616416]
46.	*hist1h4a*	Down	−1.6	0.045	Chromosome 4 open reading frame 21	[ENSECAT00000006145]
47.	*camta1*	Down	−1.6	0.039	Calmodulin binding transcription activator 1	[ENSECAT00000022451]
48.	*kcnk12*	Down	−1.7	0.033	Potassium channel, subfamily K, member 12	[XM_008530912]
49.	*phactr3*	Down	−1.7	0.035	Phosphatase and actin regulator 3	[ENSECAT00000008448]
50.	*mllt3*	Down	−1.7	0.044	Myeloid/lymphoid or mixed-lineage leukemia (trithorax homolog, *Drosophila*)	[ENSECAT00000025395]
51.	*tgfb2*	Down	−1.8	0.038	Transforming growth factor, beta 2	[ENSECAT00000017826]
52.	*scd5*	Down	−2.0	0.038	Stearoyl-CoA desaturase 5	[ENSECAT00000015537]
53.	*myf5*	Down	−2.0	0.039	Myogenic factor 5	[ENSECAT00000021416]
54.	*garem*	Down	−2.0	0.039	GRB2 associated, regulator of MAPK1	[ENSECAT00000012124]
55.	*duoxa2*	Down	−2.2	0.026	Dual oxidase maturation factor 2	[ENSECAT00000015499]
56.	*ncald*	Down	−2.2	0.044	Neurocalcin delta	[XM_005613237]
57.	*kcnmb2*	Down	−2.5	0.036	Potassium large conductance calcium-activated channel, subfamily M, beta member 2	[ENSECAT00000009305]
58.	*hla-dqb1*	Down	−5.0	0.028	Major histocompatibility complex, class II, DQ beta I	[XM_008506652]

In this paper, symbols of genes and their protein products have been presented in their original form referring to specificity of the cited paper, as lowercase italic or uppercase letters, when they refer to transcriptomic or proteomic research, respectively. In the following discussion, arrows indicate up- (↑) or down-regulation (↓) of gene expression. Validated (from microarray) or tested (*mstn*, *myog*, *igf1*) by qPCR genes are marked with hash (#).

### Real-time qPCR

**Fig. 3:**
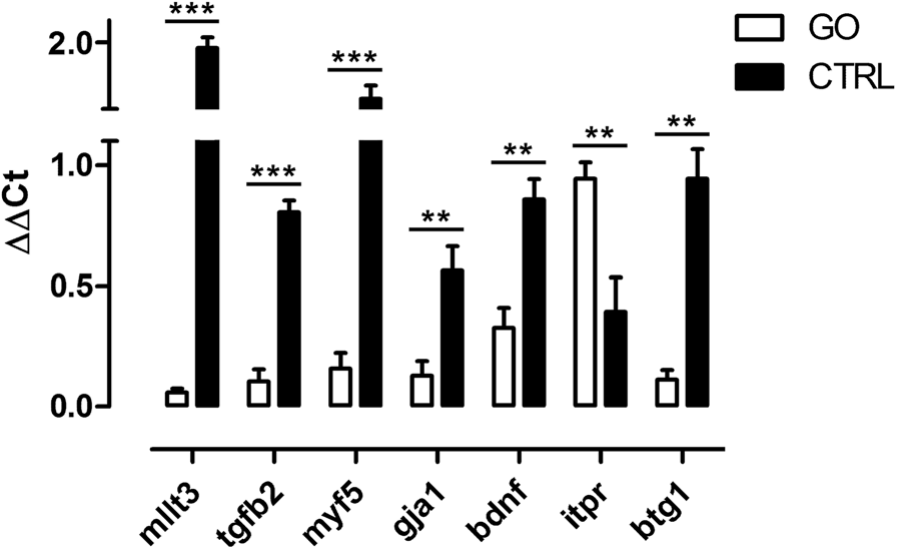
Results of qPCR validation of selected genes revealed in microarray experiment (FDR ≤ 0.05). *Asterisks* indicate the following criteria of significance: *p ≤ 0.05; **p ≤ 0.01; and ***p ≤ 0.001; (*n* = 6)

**Fig. 4:**
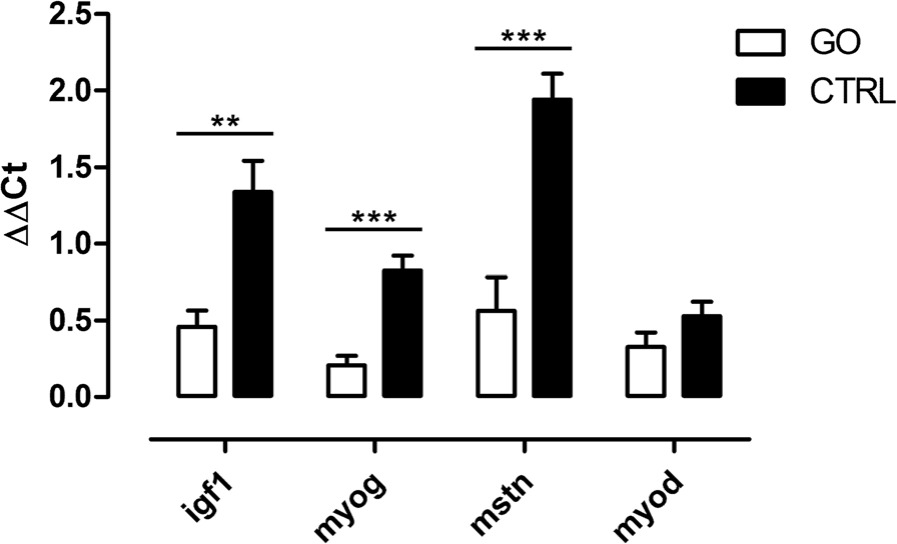
Results of qPCR analysis of additional genes important for myogenic differentiation of equine SC (FDR > 0.05). *Asterisks* indicate the following criteria of significance: *p ≤ 0.05; **p ≤ 0.01; and ***p ≤ 0.001; (*n* = 6)

According to the ontological classification and available literature, seven genes involved in the skeletal muscle development, sarcomere development, actin binding, and regulation of cell proliferation and differentiation were selected for qPCR validation. Except for gap junction protein alpha 1 (*gja1*), all expression changes from qPCR overlapped microarray results (Table 3, Fig. 3). In our study, we focused on the impact of GO on muscle development; thus, we also measured mRNA levels of three genes from muscle development profile *myog*, *igf1*, and *mstn*, which did not reach the FC ≥ 1.3 threshold (Fig. 4).

**Table 3:** Results of real-time qPCR analysis (*n* = 6)

GO vs. CTRL	Fold change	*p* value
*myf5*	−2.6	3.9E-07
*mllt3*	−2.3	1.3E-07
*tgfb2*	−1.6	3.7E-07
*gja1*	−1.3	6.8E-04
*bdnf*	−1.3	7.3E-03
*itpr2*	1.5	9.8E-03
*bdnf*	−1.3	7.3E-03
*btg1*	−1.3	2.4E-02
*myog*	−1.5	3.3E-04
*mstn*	−2.6	5.4E-04
*igf1*	−1.8	3.4E-03
*myod*	−1.1	1.6E-01

### Functional analysis

Significantly enriched gene ontologies retrieved from DAVID output files are presented in Additional file 2. In Table 4, selection of most significant biological processes is shown (EASE score <0.001), providing a comprehensive overview of important processes possibly induced by GO in differentiating ESC.

**Table 4:** Selected significantly enriched (EASE score *p* value <0.001) gene ontologies, DAVID

No.	Name	# of genes	*p* value	Genes	Bonferroni	Benjamini	FDR
1.	GO:0007517—muscle organ development	10	6.6E-8	*musk*, *cryab*, *mapk14*, *itgb1bp2*, *myf5*, *mstn*, *igf1*, *myog*, *nr2f2*, *tgfb2*	5.92E-5	5.92E-5	1.03E-4
2.	GO:0014706~striated muscle tissue development	6	7.5E-5	*musk*, *mapk14*, *myf5*, *myog*, *nr2f2*, *tgfb2*	0.06	0.03	0.12
3.	GO:0060537—muscle tissue development	6	9.4E-5	*musk*, *mapk14*, *myf5*, *myog*, *nr2f2*, *tgfb2*	0.08	0.03	0.15
4.	GO:0007519—skeletal muscle tissue development	5	1.0E-4	*musk*, *mapk14*, *myf5*, *myog*, *nr2f2*	0.08	0.02	0.16
5.	GO:0060538—skeletal muscle organ development	5	1.0E-4	*musk*, *mapk14*, *myf5*, *myog*, *nr2f2*	0.09	0.02	0.16
6.	GO:0010604—positive regulation of macromolecule metabolic process	12	2.4E-4	*mapk14*, *snca*, *myf5*, *gja1*, *mstn*, *igf1*, *abra*, *myog*, *nr2f2*, *tfap2e*, *znf423*, *tgfb2*	0.19	0.047	0.37
7.	GO:0010557—positive regulation of macromolecule biosynthetic process	10	5.8E-4	*mapk14*, *myf5*, *mstn*, *igf1*, *abra*, *myog*, *nr2f2*, *tfap2e*, *znf423*, *tgfb2*	0.40	0.08	0.89
8.	GO:0009891—positive regulation of biosynthetic process	10	8.9E-4	*mapk14*, *myf5*, *mstn*, *igf1*, *abra*, *myog*, *nr2f2*, *tfap2e*, *znf423*, *tgfb2*	0.55	0.11	1.38
9.	GO:0045941—positive regulation of transcription	9	9.9E-4	*mapk14*, *myf5*, *mstn*, *igf1*, *abra*, *myog*, *nr2f2*, *tfap2e*, *znf423*	0.59	0.10	1.5

Using Pathway Studio’s Build Pathway algorithm, we identified 116 relationships within selected entities, which are displayed in a graph view on Fig. 4. Seventy of identified relationships had the highest (third) confidence level which means that the number of references confirming this relationship was ≥3. Detected types of regulation were regulation (86), expression (28), direct regulation (1), and promoter binding (1). Fifty-five relations were positive, 22 negative, and 39 not defined. Chosen relationships are further discussed in the following chapter.

## Discussion

According to Fernyhough et al. [18], oral supplements may affect myofibers in five potential ways: (1) by direct interaction, (2) by direct influence and producing paracrine agents that affect SC, (3) by affecting SC, (4) by producing hormones that exert indirect effects in muscle cells, and (5) by protecting all cell types against oxidative damage. Although, the exact mode of GO action on muscles was not revealed, GO has been previously postulated to act through mechanisms 4 [12, 44] and 5 [17, 49]. In further discussion, we suggest that the direct interaction of GO with SC (3) may be a part of its skeletal muscle-enhancing mechanism [63]. For this purpose, genes important for myogenesis have been selected and are discussed in four functional groups.

### Ontology of differentially expressed genes

According to functional analysis, the five most significant biological processes induced by 24-h incubation of differentiating equine SC with 0.125 μmol GO are related to skeletal muscles; however, muscle organ development is the only record showing significance after FDR correction (Table 4). Moreover, GO influenced genes associated with contractile apparatus development (Additional file 2; CC and MF sheet). All this together indicates that GO, indeed, may directly influence SC activity and in vitro differentiation. Other significantly enriched processes referred to macromolecule metabolism and positive regulation of transcription (Table 4). The last has also a confirmation in enriched molecular functions: transcription regulator activity and transcription cofactor activity (Additional file 2; MF sheet). Functional analysis revealed also that GO influenced growth factor activity (Additional file 2; MF sheet). Axon and neuron projection-related genes (Additional file 2; CC sheet) could be connected with recent in vivo findings by Eslami et al. [17] who suggested that GO-induced increase in muscle strength may arise from neural adaptations without altering muscle mass.

### Interaction of GO with myogenic regulatory factors

Pre- and postnatal skeletal muscle development depends on the expression of four basic helix-loop-helix transcription factors called myogenic regulatory factors (MRFs). This group shares the ability to induce myogenic differentiation when expressed in non-muscle cells [43]. Three MRFs, *myf5*, myogenin (*myog*), and *myod*, were investigated in our study, but only two of them possessed significant changes in expression.

In adult skeletal muscle, the highest expression of *myf5* is present in committed satellite cells and decreases when progression to myocytes and myotubes occurs [4]. Likewise, in cultured equine SC, qPCR showed that *myf5* mRNA levels were highest during intensive proliferation and decreased dramatically at the onset of differentiation [25]. Compared to control cells, 24-h incubation of equine SC with GO induced down-regulation of *myf5* (↓#). According to this observation, we suggest that GO could potentially advance equine SC differentiation.

It was shown that up-regulation of MYF5 triggers the expression of myogenin (*myog*). That contributes to the withdrawal of myoblasts from the cell cycle [55]. Undetectable during proliferation, *Myog* expression level is highest when myocytes differentiate and fuse into myotubes [25] and remains at a slightly lower level in adult skeletal muscle cells [4]. Disregarded of this, additional qPCR showed down-regulation of *myog* (↓#) mRNA in experimental cells. Down-regulation of both MRFs may indicate that GO actually inhibits myogenic differentiation. Our experiment was performed, 48 h after changing proliferation medium to differentiation medium. At this stage, proliferating myocytes begin to differentiate and fuse. However, the exact cell cycle of experimental cells remains unequal and undefined. Thus, it is possible that GO has multiple ways of action and by down-regulation of *myog*, it delays terminal differentiation and simulates proliferation of equine SC. Moreover, transcription factors involved in myogenic lineage progression are not strictly acting in a linear manner but are organized in complex feedback and feed-forward networks with other muscle development genes [4]. These relationships will be discussed further in this paper.

### Genes belonging to the transforming growth factor β superfamily

Transforming growth factor β2 (*tgf-β2*) (↓#) belongs to a subset of transforming growth factor β superfamily members, which exert strong control over proliferation, migration, and adhesion of satellite cells [35]. TGF-β2 delays myoblast differentiation while increasing cellular proliferation. Molecular mechanism of TGF-β2 includes an increased rate of degradation of *myod* (Schabort et al. 2009). However, it has not been examined whether a similar relationship exists in equine skeletal muscle. In a recent study, de Mello et al. [41] showed *tgf-β2* mRNA levels were high only in proliferative C_2_C_12_ cells and began to decrease after differentiation induction. *Tgf-β2* mRNA levels were 10-fold lower after 3 days of differentiation that strongly agrees with our observations, which indicate that GO down-regulated *tgf-β2* (↓#) in equine myoblast during the third day of differentiation, when compared to control cells.

This result encouraged us to investigate another member of transforming growth factor superfamily, playing a key role in the negative regulation of muscle growth in mammals: myostatin (*mstn*). Although microarray analysis did not passed FDR correction test, additional qPCR demonstrated decreased *mstn* (↓#) mRNA level in GO vs. control cells. The deletion of myostatin in mice induces dramatic and widespread increase in skeletal muscle mass due to both muscle hypertrophy and hyperplasia [32], and it also cause double-muscling phenotype of some cattle and sheep breeds [57]. Wicik et al. [67] showed inhibitory effect of exogenous MSTN on differentiating C_2_C_12_ myoblasts.

We suggest that decreased expression of these two genes could contribute to the putative GO-mediated muscle growth enhancement, by increasing differentiation process. However, this needs to be reconciled with decreased expression of differentiation marker *myog*.

### Other genes involved in cell proliferation and differentiation processes

Our interest was also focused on genes involved in cell proliferation and differentiation, described in the literature as important for myogenesis.

Mitogen-activated protein 14 (*mapk*14) (↑) also called p-38α belongs to one of the major regulators of gene transcription and metabolism in response to oxidative, energetic, and mechanical stress in skeletal muscle. Chronic activation of MAPK14 signaling pathway has been implicated in the development of adaptive and maladaptive response in skeletal muscle pathologies, such as diabetes [7] and protein catabolism [53], and physiological states, such as growth and differentiation [34]. Mapk14 expression coincides with *myog* expression during differentiation [8], which could potentially indicate the role of GO in muscle differentiation; however, in our study, expression of this genes was opposite.

**Fig. 5:**
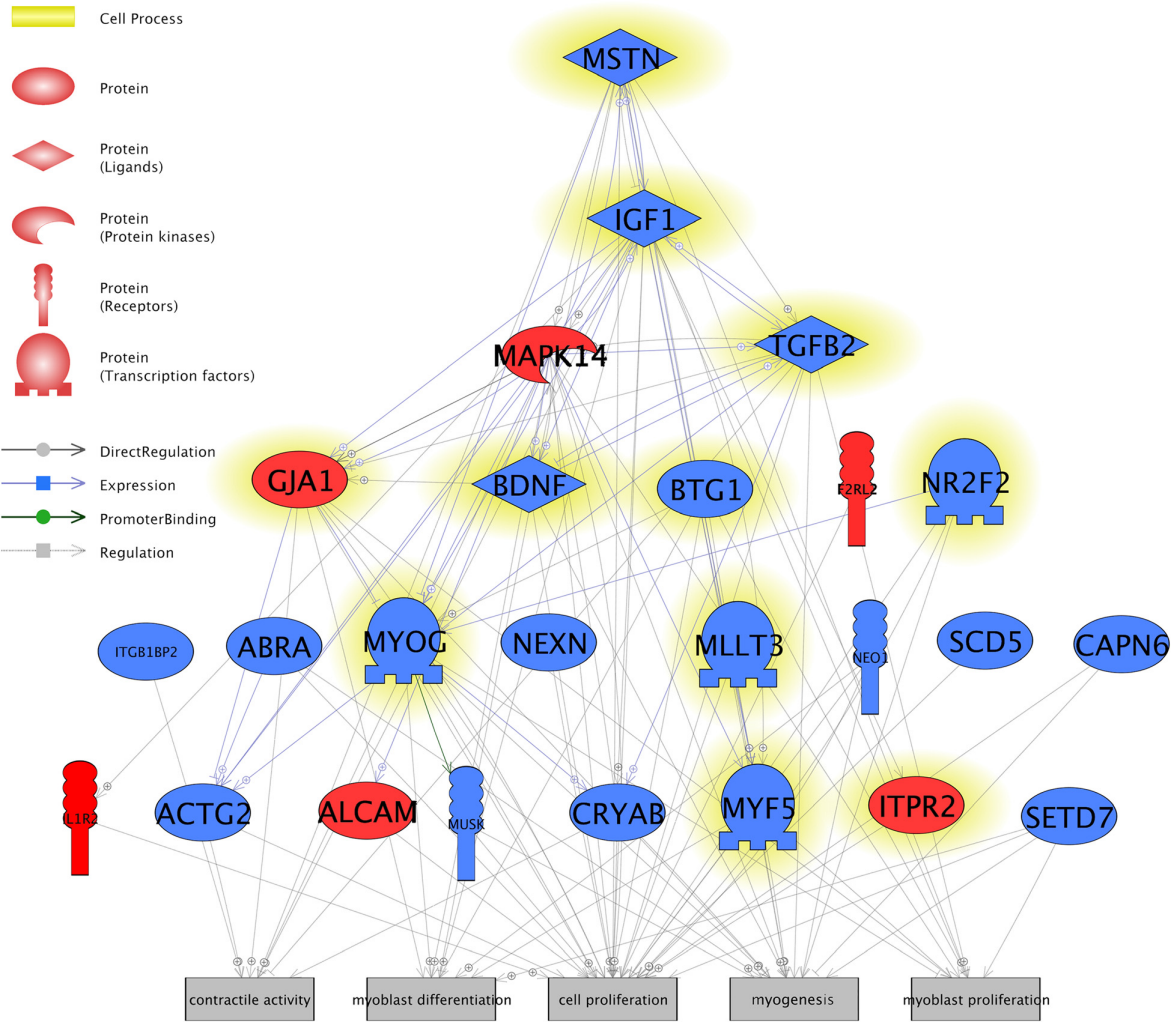
Relationships between differentially expressed genes, based on Pathway Studio. Genes are marked with *red* and *blue colors* for up- and down-regulation, respectively. The pathway was enriched with myogenesis-related biological processes and additional qPCR-tested genes of muscle development profile (*mstn*, *igf1*, *myog*). Genes validated or tested by qPCR are *highlighted*

According to the qPCR analysis, exposition of SC to GO induced significant down-regulation of insulin-like growth factor 1 (*igf-1*) (↓#). *Igf-1* is, up to date, the only known growth factor positively regulating proliferation and differentiation of these cells [60]. IGF-1 can indirectly affect satellite cells, by increasing the expression of *myf5*, *myog*, and *myod*, and embryonic myosin heavy chain [70] and decreasing expression of *mstn* [56]. Analysis of the relationships between the abovementioned genes places *igf-1* on the top of the signaling cascade hypothetically induced by GO (Fig. 5). Thus, it may be suggested that GO decreases expression of MRF through the inhibition of *igf-1* signaling. These findings are in contrast with presumed abilities of GO.

Haddad and Adams [24] indicated that intact signaling through MAPK is necessary for the development of IGF-I-induced skeletal muscle hypertrophy in vivo. However, in our study, this relationship was not observed, as indicated by different changes in the expression of these two molecules. In contrast, Trendelenburg et al. [66] showed that pro-inflammatory cytokines may inhibit myoblast growth via p38 signaling activity. This observation corresponds with up-regulation of interleukin 1 receptor type II (*il1r2*) (↑) expression and MRF’s down-regulation in our culture cells, suggesting that GO can activate stress-related signaling in cultured cells.

Another gene deserving attention is *gja1* (↓#) gene encoding gap junction protein alpha 1—component of intercellular channels that provides a route for the diffusion of low molecular weight particles from cell to cell. Except for that, GJA1 affects cell proliferation and differentiation in various tissues. Although not present in normal adult skeletal muscle, GJA1 is necessary during regenerative myogenesis in rodents [1, 21]. The relationship between *gja1* and MRF expression was observed in the L6 myogenic cell line; pharmacological gap junction blockers were found to inhibit myogenin expression and myotube formation, reversibly [3]. Likewise, delayed expression of *myog* was found in myoblast cultures prepared from mice with an inducible deletion of *gja1* [1]. Similar function in differentiating myoblasts was reported for B cell translocation gene 1 (*btg1*) (↓#). The *btg1* overexpression inhibited proliferation of several cell lines including NIH3T3, PC12, and QM7 quail myoblast lines [52]. Therefore, it was initially believed that these proteins possess antiproliferative properties. However, other studies demonstrated that *btg1* up-regulation is crucial at the onset of myoblast differentiation [54]. More recently, Busson et al. [9] described the molecular basis of *btg1* action and proved that it stimulates the activity of not only MRFs but also nuclear receptors already known as positive myogenic regulators (T3 and all-*trans* retinoic acid receptors).

From all these studies, it becomes evident that the outcome of *gja1* and *btg1* expression in muscle cells is associated with the onset of differentiation while irreversible withdrawal from the cell cycle occurs. Thus, the context of the signal is essential for understanding their role in GO signaling. Taking into account that GO was added to differentiating myoblasts, *gja1* and *btg1* down-regulation indicate that GO may decrease equine myoblast fusion and could suggest pro-proliferative action of *btg1* and *gja1* in equine SC exposed to GO, which agrees with the reduced levels of MRF’s expression.

Herein, we demonstrate down-regulation of brain-derived neurotrophic factor (*bdnf*) (↓#). *Bdnf* is a member of the mammalian family of neurotrophins, studied for their roles in the nervous system function [13]. Further expression-profiling studies have shown that *bdnf* is differentially expressed in skeletal muscle under various physiological and pathological conditions [11] but *bdnf* expression pattern in certain physiological states remained elusive. Mousavi and Jasmin [45] showed that *bdnf* expression during myogenic differentiation has been significantly reduced. Moreover, siRNA knockdown of *bdnf* expression evoked precocious differentiation of myoblasts. This effect was inhibited by the administration of recombinant *bdnf*. However, more recently, Clow and Jasmin [13] indicated that instead of its inhibitory role, bdnf^−/−^ mice demonstrated impaired induction of several molecular markers of regeneration, including *pax7*, *myod*, and *myog* when proliferating myoblasts began to differentiate and fuse to repair damaged myofibers. The role of *bdnf* remains questionable; however, attenuated MRF’s expression in our culture may confirm that expression of this gene is required for normal myogenic differentiation. This finding can also be applied to the next specified molecule down-regulated by GO: αβ-crystallin (*cryab*), (↓) a small heat shock protein involved in preventing protein aggregation. Expression of *cryab* was induced selectively in myoblasts during an early stage of their differentiation in vitro [33]. Moreover, mice lacking αβ-crystallin died prematurely with extensive muscle wastage [59] which indicates its essential role during myogenesis. Indeed, the αβ-crystallin promoter contains canonical skeletal muscle-specific E-box element that binds MRFs [20]. On the other hand, overexpression of *cryab* inhibits myogenic differentiation by negatively regulating differentiation-related apoptosis, inhibiting caspase-3 activation and altering *myod* levels [33, 59].

Next, gene affected by the presence of GO which deserves attention is nuclear receptor 2F2 (*nr2f2*) (↓#) alias chicken ovalbumin upstream promoter transcription factor II (*coup-tfII*). It encodes nuclear orphan receptor of the steroid-thyroid hormone receptor superfamily. Recently, growing evidence suggests the pivotal role of COUP-TFs in mesenchymal stem cell (MSC) regulation and development [69]. As illustrated both in vitro and in vivo, loss of COUP-TFII reprograms the precursors by shifting the cell identity to osteogenic and myogenic lineages at the expense of adipogenic and chondrogenic programs, supporting the notion that COUP-TFII is essential for fate choice and commitment [68]. The molecular mechanism by which COUP-TFII affects MSC development is, at least partially, through the down-regulation of Wnt cascade substrate: Wnt10b. This phenomenon resulted in reduced fat tissue and enhanced glucose tolerance and insulin sensitivity, as well as increased bone mineralization and muscle mass in COUP-TFII-deficient mice [68]. Moreover, previous reports indicated that COUP-TFII inhibits myogenesis by the transcriptional and posttranscriptional regulation of *myod* [2, 46]. In conclusion, down-regulation of this gene may indicate that GO stimulates in vitro myogenesis.

Finally, in our study, increased expression (↑) of activated leukocyte cell adhesion molecule (*alcam*), also known as cluster of differentiation 166 (CD166), has been detected. *Alcam* plays an important role in cell proliferation and the differentiation of mesenchymal tissues in multiple species [61]. Moreover, *alcam* may increase cell survival in several tumor types as well as monocytes [61]. These abilities may result from the antiapoptotic capacity of this protein [39]. Other authors speculate that ALCAM-ALCAM interactions, in cooperation with other adhesion molecules, facilitate stroma progenitor cell contact, resulting in proliferation of progenitor cell subsets [47]. It may be presumed that expression of *alcam* could mediate GO action on SC, but the functional roles in muscle physiology of this marker remains unknown and needs further investigation.

Other genes positively regulating muscle differentiation, whose expression was changed by GO, include neogenin (*Neo1*) (↓), actin-binding Rho-activating protein (*Abra*) (↓), actin γ-2 (*actg2*) (↓), integrin beta-1-binding protein 2 (melusin 2, *Itgb1bp*2) (↓), muscle-specific tyrosine kinase receptor (*Musk*) (↓), nexilin (*nexn*) (↓), histone H3-K4 methyltransferase (*setd7*) (↓), and calpain 6 (*capn6*) (↓)-myogenesis-suppressing gene [5, 14, 27, 36, 37, 42, 64, 65]. Down-regulation of these genes may suggest that 24-h incubation with 0.125 μM of GO decreased differentiation of equine satellite cells.

### Other gamma-oryzanol treatment-related genes

GO has been reported to possess strong antioxidant activity; thus, it is postulated that GO might enhance endurance and muscle-building capacity by hindering the production of free radicals, which in theory could lessen muscle exhaustion and fatigue in reaction to anaerobic exercise [17]. Consistent with this, significantly lower post-exercise total antioxidant status and thiobarbituric acid-reactive substance level were observed in racing horses receiving GO compared to horses from other groups [49]. GO’s influence on oxidative stress-related genes has been shown in other tissues [30, 31]. However, in our study, gene ontology analysis did not show any significant involvement of differentially expressed genes in antioxidative processes. It will be important in future studies to determine if antioxidant activity is involved in the protective effects of GO under oxidative stress conditions.

Lowered expression of stearoyl-CoA desaturase and isoform 5 gene (*scd5*) (↓) as well as of *coup-tfII* (↓) may reflect positive influence of GO on energy homeostasis in skeletal muscle. As mentioned before, *Coup-tfII*-deficient mice demonstrated reduced fat tissue and enhanced glucose tolerance and insulin sensitivity [68]. Likewise, SCD deficiency is evidenced to activate metabolic pathways which promote β-oxidation and decrease lipogenesis both in the liver and skeletal muscles. SCD mutation results also in general changes in the expression of genes involved in lipid metabolism. SCD1-deficient mice have increased energy expenditure and reduced body adiposity and are resistant to diet-induced obesity (Dobrzyn and Ntambi 2005). These results may confirm GO utility in the treatment of diabetes (Ohara et al. 2009) as well as positive effect on lean body mass gain.

## Conclusions

**Fig. 6:**
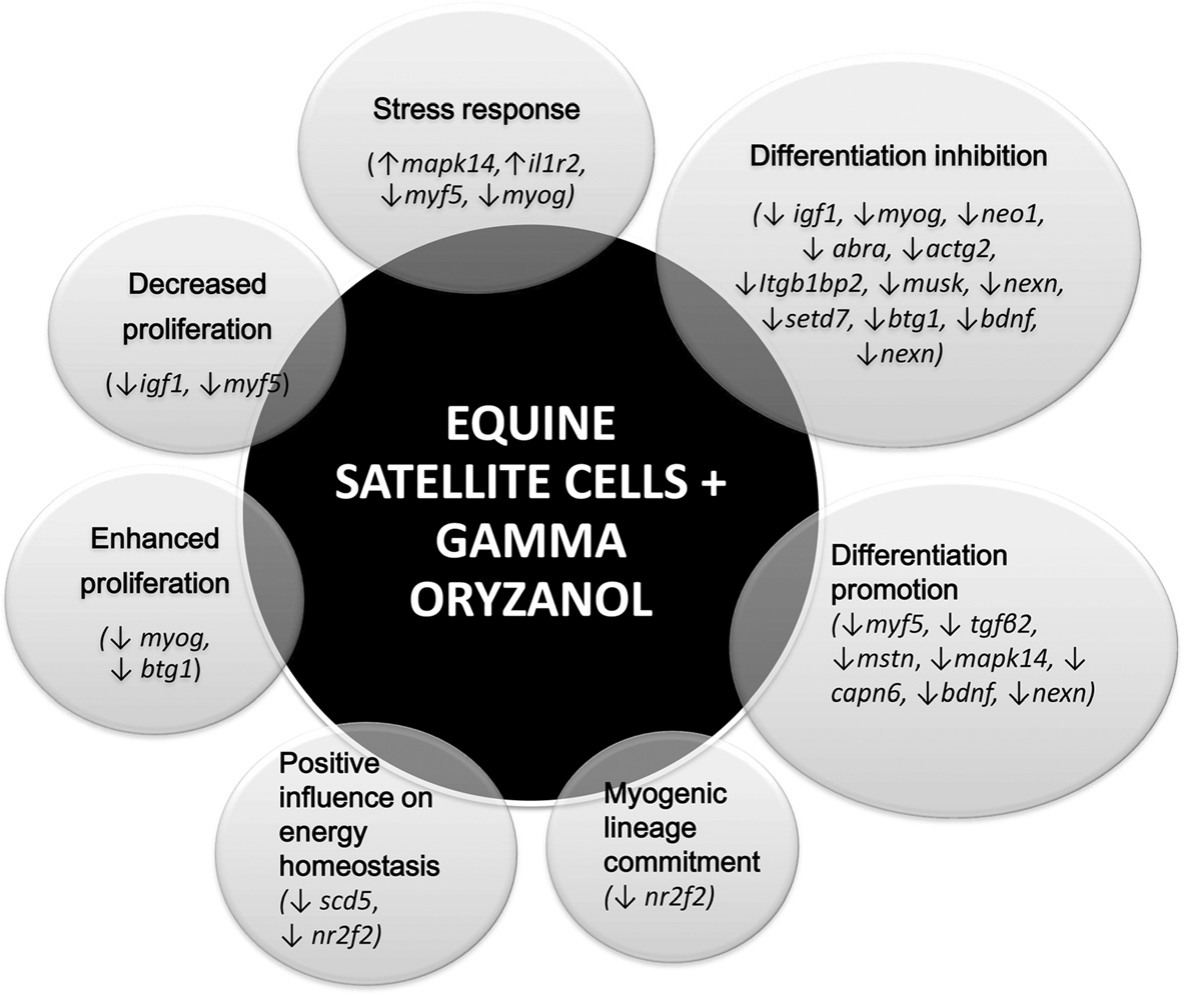
Processes reinforced in equine satellite cells incubated with 0.125 μM of GO for 24 h

In conclusion, we demonstrated for the first time that GO can affect the transcriptomic profile of equine satellite cells in vitro. Many of differentially expressed genes are reported to be crucial for skeletal muscle development. Analysis of expression changes proves that various processes are reinforced by GO in equine SC (Fig. 6); however, inhibition of myoblast differentiation is prevailing observation. Incubation with GO was performed at the onset of differentiation; thus, decreased expression of differentiation markers may indicate that GO delays this process in cultured equine SC that may contribute to increased field of cells that will later differentiate into myotubes. This could be explained in further investigation of GO’s influence on satellite cells in proliferation stage. Beyond this, 24-h incubation with GO induced different expression of genes responsible for stress response, increased myogenic lineage commitment, cell proliferation and differentiation, and finally positive influence on energy homeostasis. Our study may confirm putative muscle-enhancing abilities of GO; however, analysis of changes in gene expression did not give equivocal results. Gene transcription is only one step in the regulatory pathway that leads to the functional protein synthesis. Our results encourage for investigation of GO-skeletal muscle relation in proteomic and biochemical level in the future. The collective role of GO in skeletal myogenesis remains unclear and needs further investigation.

## Compliance with ethics guidelines

This article does not contain any studies with human or animal subjects performed by the any of the authors.

## Additional files

Additional file 1:
**Full list of differentially expressed transcripts, gamma-oryzanol vs. control.** (XLS 55.0 kb) Additional file 2:
**Full list of significantly enriched (EASE score** ***p*** **value ≤0.05) gene ontologies (DAVID).** (XLS 42.5 kb)
